# Endogenous tagging of multiple cellular components in the sea anemone *Nematostella vectensis*

**DOI:** 10.1073/pnas.2215958120

**Published:** 2022-12-27

**Authors:** Alexandre Paix, Soham Basu, Peter Steenbergen, Rajwinder Singh, Robert Prevedel, Aissam Ikmi

**Affiliations:** ^a^Developmental Biology Unit, European Molecular Biology Laboratory, Heidelberg 69117, Germany; ^b^Cell Biology and Biophysics Unit, European Molecular Biology Laboratory, Heidelberg 69117, Germany; ^c^Epigenetics and Neurobiology Unit, European Molecular Biology Laboratory, Monterotondo 00015, Italy

**Keywords:** Cnidaria, CRISPR/Cas9, HDR, knock in, protein dynamics

## Abstract

The cnidarian *Nematostella vectensis* has developed into a powerful model system to study the mechanisms underlying animal development, regeneration, and evolution. However, despite the significant progress in the molecular and genetic approaches in this sea anemone, endogenous protein tagging is still challenging. Here, we report a robust method for knock in for *Nematostella* using CRISPR/Cas9. As an outcome, we generate endogenously tagged proteins that label core molecular components of several cellular apparatus, including the nuclear envelope, cytoskeleton, cell adhesion, endoplasmic reticulum, cell trafficking, and extracellular matrix. Using live imaging, we monitor the dynamics of vesicular trafficking and endoplasmic reticulum in embryos, as well as cell contractility during the peristaltic wave of a primary polyp. This advancement in gene editing expands the molecular tool kit of *Nematostella* and enables experimental avenues to interrogate the cell biology of cnidarians.

Cnidarians are morphologically simple animals with a body plan composed of two epithelial layers (1). This relative simplicity of cnidarian tissue architecture provides an ideal context to study the molecular basis of biological processes. The advent of genome-editing tools has enabled achieving gene knockouts in several cnidarian species (2–4). However, protein visualization in cnidarians still relies primarily on microinjection of mRNA or random insertion of transgene overexpressing fluorescently tagged proteins, which can lead to problems such as false localization, protein misfolding, and artifactual interactions. Furthermore, immunostaining experiments are primarily used with fixed specimens, restricting access to the dynamic changes in protein localization. To overcome these limitations, endogenous protein tagging with fluorescent markers is a valuable method to recapitulate physiological gene expression and protein localization in vivo. Using CRISPR/Cas9 (5), homology-directed repair (HDR)–based methods for generating knock-in (KI) lines have been described in two cnidarian species, *Nematostella* and *Hydractinia* (4, 6–9)*.* However, only a few KI cnidarian lines have been reported, reflecting the current difficulties of achieving protein tagging at the endogenous locus.

## Results and Discussion

**Fig. 1.**
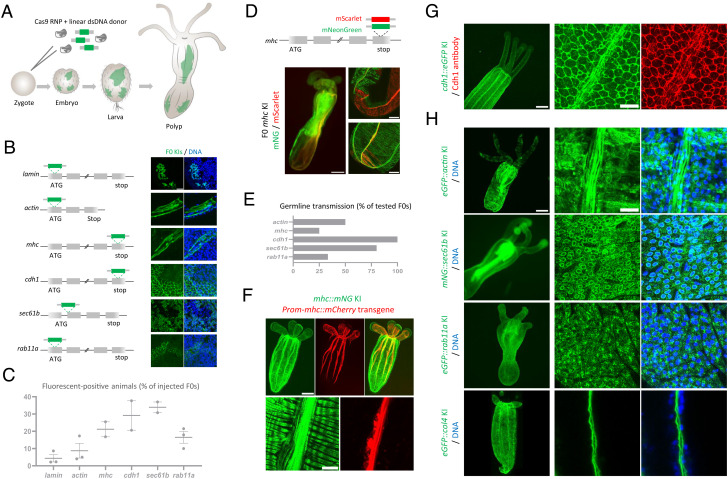
(*A*) Schematic representation of KI generation. (*B*) Schematic representation of 6 targeted loci. Images showing the subcellular localization of the tagged proteins in F0s (4 to 12 F0s were imaged for each gene). (Scale bar, 10 µm.) (*C*) Plot showing mean and SEM of the percent of injected embryos expressing the fluorescent protein (n ≥ 2 injection sessions). *Lamin*: 2.2% (5/223), 2.3% (6/259), and 8.6% (2/23); *actin*: 4.0% (4/100), 5.0% (16/319), and 17.3% (4/23); *mhc*: 16.8% (16/95) and 25.5% (12/47); *cdh1:* 20.6% (27/131) and 37.7% (23/61); *sec61b*: 30.8% (46/149) and 37.0% (83/224); and *rab11a*: 10.0% (19/190), 17.9% (28/156), and 21.5% (92/426). (*D*) Schematic representation of the KI of mNG (green) and mScarlet (red) in the *mhc* locus. Whole view of a two-color positive KI (F0) and confocal images showing the distribution of mNG and mScarlet proteins. (Scale bars, 100 µm and 30 µm.) (*E*) Plot showing the percentage of F0s with germline transmission of the KI. *Actin*: 50.0% (2/4), *mhc*: 25.0% (1/4), *cdh1:* 100% (4/4), *sec61b*: 80.0% (4/5), and *rab11a*: 33.3% (2/6). (*F*) Primary polyp expressing *mhc::mNG* and the mCherry transgene driven by the *mhc* promoter. (Scale bar, 100 µm.) Note the lack of expression of the transgene in the circular muscle. (Scale bar, 10 µm.) (*G*) *cdh-1::eGFP* primary polyp. (Scale bar, 100 µm.) Immunofluorescence showing the overlap between total Cdh1 protein and endogenously tagged Cdh1 in a heterozygous *cdh1::eGFP* animal. (Scale bar, 10 µm.) (*H*) Whole view of F1s/F2s expressing the indicated KI alleles and showing the detailed localization of tagged proteins. (Scale bars, 100 µm and 10 µm.)

To maximize the efficiency of gene KI in *Nematostella*, we used Cas9 protein and chemically synthesized sgRNA combined with a PCR-generated linear repair donor bearing short homology arms (30 to 40 bp) and inserted in the vicinity of the Cas9-induced double-strand break (DSB) (10). Using microinjections of fertilized eggs, we tested six loci for fluorescent protein tagging (Fig. 1*A*). The targeted proteins mark key cellular components, including the nuclear envelop (lamin/Nt), cytoskeleton (actin/Nt and myosin heavy chain—Mhc/Ct), adhesion (cadherin 1—Cdh1/Ct), and cellular trafficking (Sec61b/Nt and Rab11a/Nt) (Fig. 1*B*). Between 2.2 and 37.7% of injected embryos expressed the fluorescent protein (Fig. 1*C*). Examination of these embryos showed the expected expression patterns, and the subcellular localization of the tagged proteins was confirmed by confocal microscopy in a few animals, suggesting the in-frame KI of the repair donors at the targeted loci (Fig. 1*B*). Given the high rate of fluorescent-positive embryos in *mhc*, we tested biallelic targeting of this gene using two fluorescent proteins, mNeonGreen (mNG) and mScarlet (Fig. 1*D*). Approximately one third of fluorescent-positive embryos had expression of both markers. However, we detected a minor overlap between mNG-positive and mScarlet-positive cells, indicating rare biallelic HDR in single cells. This condition might be due to nonhomologous end-joining (NHEJ)–mediated repair causing the loss of the Cas9-cutting site in one allele.

**Fig. 2.**
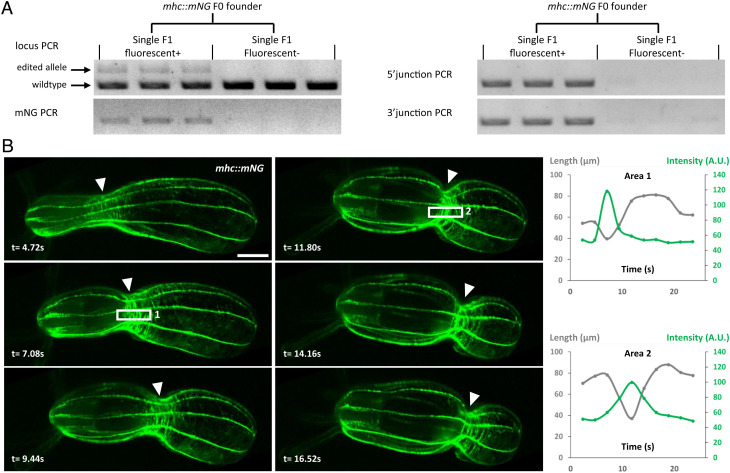
(*A*) Agarose gel showing PCR genotyping of single F1s from the *mhc::mNG* founder. (*B*) Live imaging of a peristaltic contraction in the *mhc::mNG* polyp (Movie S1). (Scale bar, 100 µm.) Arrowheads indicate the localization of the contraction ring of the peristaltic wave. Time in seconds. Quantification of the Mhc::mNG signal across the peristaltic ring: two examples of area quantified are shown (1 and 2). Signal intensities (arbitrary units, A.U.) were measured in the area delimited in the white rectangles; lengths were measured between surrounding rows of longitudinal muscle.

To assess for germline transmission, we focused on growing a subset of F0 polyps that exhibit broad expression of the fluorescent signal and tested between four and six animals for each gene. By crossing potential founders with wild-type animals, we obtained germline transmission for all targeted loci except *lamin*. This lack of germline transmission might be explained by the deleterious effects of endogenous tagging of *lamin* combined with the generation of knockout alleles. For the other targeted loci, between 25 and 100% of tested animals were founders (Fig. 1*E*), with positive F1s representing in most cases 50% of each progeny. As a result, we established stable F1 lines for *actin*, *mhc*, *cdh1*, *sec61b,* and *rab11a*. In addition, we generated a KI line for *collagen IV*—*col4* (Nt) to mark the basement membrane (Fig. 1 *F*–*H*). Molecularly, scarless insertions were detected in all tested F1s using sequencing (n = 41 F1s from 12 edited F0s for *actin*, *mhc*, *cdh1*, *sec61b, rab11a*, and *col4*). These data suggest that PCR-amplified donors generate precise KIs. Additionally, we did not detect any random insertions of the donor PCR in fluorescent-negative F1s (n = 35) (Fig. 2*A*), while several of them showed NHEJ repair.

Next, we examined the expression and localization of endogenously tagged proteins (Fig. 1 *F*–*H* and Movies S1–S6). Consistent with the Cdh1 immunostaining data (11), Cdh1::eGFP was localized at the apical and basal junctions of both body wall layers and was weakly expressed in the epidermal tissue of the tentacles (Fig. 1*G*). A prominent signal of Cdh1::eGFP was also detected along the longitudinal muscles in primary polyps, likely mediating strong adhesion between contractile cells. The mNG::Sec61b protein showed the expected localization at the endoplasmic reticulum (ER), surrounding the nuclear envelope (Fig. 1*H*). In embryonic cells, mNG::Sec61b clearly labeled the reticulate network of ER in the cell cortex and its connection to the nuclear envelope with cytoplasmic tubules (Movies S2–S4). Live imaging of early embryos showed that the localization of mNG::Sec61b is dynamic during the cell cycle (Movie S5). The mNG::Rab11 protein formed puncta localized mostly on the cell surface (Fig. 1*H* and Movie S6). These puncta displayed dynamic movement with an average velocity of 0.25 μm/s, probably reflecting Rab11a-mediated vesicle trafficking. In primary polyps, eGFP::Col4 showed widespread expression and formed two thin extracellular sheets of basement membrane for both the epidermis and gastrodermis (Fig. 1*H*). For cell contractility, Mhc::mNG was expressed in circular and longitudinal muscles with a dominant localization at their basal myofilaments (Fig. 1*F*). This expression pattern partially overlapped with the reporter transgene driven by the *mhc* promoter (12), which showed restricted expression in the longitudinal muscles with no detected expression in circular muscles. This result emphasizes the importance of the KI approach for gene expression analysis. Using a custom-made light microscope (13), we tracked the dynamics of Mhc::mNG localization throughout a peristaltic wave (Fig. 2*B* and Movie S1). Consistent with the constriction pattern, we detected an enrichment of Mhc::mNG at the level of the peristaltic wave. Intriguingly, the spatial pattern of Mhc::mNG formed periodic bands instead of a uniform distribution.

This KI method is simple, requiring no cloning of the donor construction, enabling us to tag several genes with reduced, if any, random integration of the donor DNA. By injecting 300 to 400 *Nematostella* zygotes and selecting a few fluorescent-positive animals with broad expression, it is possible to obtain germline transmission from four to six F0s. Although it is still unclear which HDR repair mechanisms are implicated, short homology arms may be used to repair DSB using a conserved HDR mechanism called synthesis-dependent strand annealing (SDSA) (14). In SDSA, genomic 5'-ends at the DSB are resectioned by exonucleases, and single-strand 3'-ends anneal with the donor to start replication and integration of the donor DNA. As the generated KI lines label key cellular components, they will be a great resource for the cnidarian community. We believe this method can be used in the future to generate reporter lines for other fundamental biological processes to explore the fascinating biology of *Nematostella* and other cnidarians.

## Materials and Methods

Embryo microinjection, CRISPR/Cas9 KI design, imaging, and genotyping were performed as previously described (4, 10). CRISPR/Cas9 KI mix consisted of 1.3 to 1.5 μg/μL Cas9 protein, 0.6 μg/μL sgRNA, 0.24 to 0.26 μg/μL repair donor, and 5 to 10 μg/μL fluorescent dextran. Detailed descriptions are provided in *SI Appendix*.

## Supplementary Material

Appendix 01 (PDF)

Movie S1. **Live imaging of a *mhc::mNG* contracting polyp.** Non-anesthetized primary polyp. Data shown in Figure 2B. Scale bar = 100μm.

Movie S2. **Live imaging of a *mNG::sec61b* embryos.** Z-stack of a 32-cell stage embryo. Scale bar = 100μm.

Movie S3. **Live imaging of a *mNG::sec61b* embryo.** 32-cell stage embryo (same as Movie S2). The focus is at the level of the cell cortex and shows ER tubules and patches. Note that some bleaching occurs. Scale bar = 10μm.

Movie S4. **Live imaging of a *mNG::sec61b* embryo.** Gastrula stage, with gastrulation hole at the center. Scale bar = 20μm.

Movie S5. **Live imaging of a *mNG::sec61b* embryo.** Low resolution of dividing blastomeres (128-cell stage embryo). Scale bar = 20μm.

Movie S6. **Live imaging of a *eGFP::rab11a* embryo.** 8-cell stage embryo. eGFP::Rab11a forms puncta, possibly corresponding to trafficking vesicles, and some of them were manually tracked (lines). Scale bar = 5μm.

## Data Availability

All KI lines are available upon request. sgRNA and primer and donor sequences are available in *SI Appendix* and DRYAD repository (https://doi.org/10.5061/dryad.63xsj3v5s).
