# Supplementary material for: Endogenous tagging of multiple cellular components in the sea anemone Nematostella vectensis
Source: Proc Natl Acad Sci U S A. 2022 Dec 27;120(1):e2215958120. doi: 10.1073/pnas.2215958120 (PMC9910423; doi:10.1073/pnas.2215958120)
Supplement: Supplementary file 1 — Appendix 01 (PDF) [file pnas.2215958120.sapp.pdf]

SUPPORTING INFORMATION

Endogenous tagging of multiple cellular components in the sea anemone *Nematostella vectensis*

Alexandre Paix, Soham Basu, Peter Steenbergen, Rajwinder Singh, Robert Prevedel and Aissam Ikmi

Authors for correspondence: alexandre.paix@embl.de and aissam.ikmi@embl.de

SUPPORTING MATERIAL AND METHODS

Animals

Adult *Nematostella* were maintained in a circulating system with 12 parts per thousand (ppt) artificial seawater (1/3-SW) (sea salt, instant ocean) at 17 °C in the dark. Adults were induced for spawning every 3 weeks using a light box with a temperature of 28 °C and light intensity of 250–300 lumen per square foot for approximately 6h. Spawning occurred within 3–4h after a cold-water change (17 °C) (1).

Repair donors and guide RNAs

Repair donors and guide RNAs were designed to have insertion as close as possible to the Cas9 DSB, with homology arms between 30-40bp, at least one homology arm adjacent to the DSB, and if applicable, the sequence between the Cas9 DSB and the insert recoded with silent mutations (2). 5'-Biotin modification is added to the repair donor to potentially prevent the formation of DNA concatemer in vivo (3).

Targeted loci were identified on SIMRbase (4). Transcript models used for design were: nv2m00015943.1.mRNA.1 (*lamin*), nv2m00013404.1.mRNA.1 (*actin*), nv2m00013141.1.mRNA.1 (*mhc*), nv2m00018397.1.mRNA.1 (*cdh1*), NVEC200_000975_1.1 (*sec61b*), nv2m00005163.1.mRNA.1 (*rab11a*), nv2m00020238.1.mRNA.1 (*col4*, note that the transcript model was manually corrected for exon splicing). For Col4 Nt fusion, eGFP was inserted after the signal peptide coding sequence. For Actin Nt fusion, no guide RNA could be found near the start codon, and the guide RNA used was cutting at the beginning of a region coding for an Actin domain. To avoid disrupting this domain after tagging, the repair donor contains the recoded beginning of the Actin domain to maintain its integrity after eGFP insertion.

Together with the reported fluorescent reporter, some repair donors contain other sequences such as linker or antigenic peptide tags. Inserts are: eGFP (714bp) or mNG (705bp) for Lamin Nt tagging, eGFP+VAALVVD (735bp) for Actin Nt tagging, Linker::3xFlag::mNG (798bp) or Linker::3xFlag::mScarlet (786bp) for Mhc Ct tagging, eGFP (714bp) for Cdh1 Ct tagging, mNG (705bp) for Sec61b Nt tagging, eGFP (714bp) or mNG (705bp) for Rab11a tagging, eGFP::Linker::HAtag::Linker (804bp) for Col4 Nt tagging.

Distances between Cas9 cut (3bp upstream PAM) and insertion site are: 1bp (*lamin* and *actin*), 2bp (*mhc*), 4bp (*cdh1*), 3bp (*sec61b* and *rab11a*), 2bp (*col4*).

Homology arms lengths are: 35bp/35bp (*lamin*), 33bp/33bp (*actin*), 33bp/37bp (*mhc*), 34bp/33bp (*cdh1*), 36bp/36bp (*sec61b*), 33bp/33bp (*rab11a*), 34bp/34bp (*col4*).

For each KI, forward and reverse PCR primers (5' to 3') to amplify the PCR donor are: 5'Biotin-GCTTTAATTCGGCTAAAATGGCAACAGCGACCAAAGTGAGTAAAGGAGAAGAAC and 5'Biotin-CGGAGTCTTTGGAGTGGAAGATGAGCTAGCAGGACTCTTGTACAGCTCGTCCATG (eGFP, *lamin*); 5'Biotin-GCTTTAATTCGGCTAAAATGGCAACAGCGACCAAAGTCTCCAAAGGGGAGGAAG and 5'Biotin-CGGAGTCTTTGGAGTGGAAGATGAGCTAGCAGGACTCTTGTAGAGTTCGTCCATTC (mNG, *lamin*); GTGTGACGACGACGTTGCTGCTCTGGTTGTTGACGTGAGCAAGGGCGAGGAGC and GGCGAAACCGGCCTTGCACATACCGGAGCCATTATCTACCACTAACGCTGCTACCTTGTACAGCTCGTCCATG, followed by PCR with 5'Biotin-GTGTGACGACGACGTTGCTG and 5'Biotin-GGCGAAACCGGCCTTGCAC (eGFP+VAALVVD, *actin*); 5'Biotin-CAAGCAGCTACCGAAGTGCTTCCTCAACCTCATTATCTAGCGGACCTTCAGGTAG and 5'Biotin-CAAAATTATTAAATTCTTGGGTAGATTATTCGTCATCCTTGTAGAGTTCGTCCATTC (Linker::3xFlag::mNG, *mhc*); 5'Biotin-CAAGCAGCTACCGAAGTGCTTCCTCAACCTCATTATCTAGCGGACCTTCAGGTAG and 5'Biotin-CAAAATTATTAAATTCTTGGGTAGATTATTCGTCATCCTTGTACAGCTCGTCCATG (Linker::3xFlag::mScarlet, *mhc*); 5'Biotin-CAAACTTGCAAAGCTGTATGAAGACGTGGATGAGGTGAGCAAGGGCGAGGAGC and 5'Biotin-CTCCATAAATGTCAATTTTAAAAATCCCCTATTAtcACTTGTACAGCTCGTCCATG (eGFP, *cdh1*); 5'Biotin-GAATATCTGCTTGAACTATAGGTTGAAAAGTCGCCTGTCTCCAAAGGGGAGGAAG and 5'Biotin-CTTAGAAGGCGATCTCCCGGCCCCGACAGCGGTGGATGACTTGTAGAGTTCGTCCATTC (mNG, *sec61b*); 5'Biotin-GGATTCTAGCTGTGTATAACTTGCGTTACGATTATGGTGAGCAAGGGCGAGGAGC and 5'Biotin-GAAAAGATAGTCGTATTCATCGTCTTTCGTCCCCTTGTACAGCTCGTCCATG (eGFP, *rab11a*); 5'Biotin-GGATTCTAGCTGTGTATAACTTGCGTTACGATTATGGTCTCCAAAGGGGAGGAAG and 5'Biotin-GAAAAGATAGTCGTATTCATCGTCTTTCGTCCCCTTGTAGAGTTCGTCCATTC (mNG, *rab11a*); 5'Biotin-

TAAGTGCCAAGGCTGCCAGGCGTGTGTTCCCAGAGTGAGTAAAGGAGAAGAAC and 5'Biotin-GTTTTTACTCACCCGATCGCCTTTCTCACCAATACAACTAGTTCTAGAGCGGCCG (eGFP::Linker::HAtag::Linker, *col4*).

Plasmids (containing only the inserts) used for PCR are: pAP27 (submitted to Addgene), pAP28 (submitted to Addgene), pAP625 (Addgene #70051), pAP683 (Addgene #99488), pAP1698 (Addgene #105242).

Genomic sequence targeted by sgRNA (20nt upstream a NGG PAM, 5' to 3') are: AGATGAGCTAGCAGGACTTT (*lamin*), GCTGCTCTGGTTGTTGACAA (*actin*), AGATTATTCGTCATCTAATG (*mhc*), AGACGTGGATGAGTAGTAAT (*cdh1*), GCCCCGACAGCGGTGGATGA (*sec61*b), TAACTTGCGTTACGATTATG (*rab11a*), GCGTGTGTTCCCAGATGTAT (*col4*).

Repair donors were synthesized by PCR using primers (Sigma, salt-free, 100µM in H2O) annealing with the extremities of the inserts and contain sequence homologous to each side of the Cas9 cut (homology arms) and if applicable, sequence between Cas9 cut and insertion site (2). If necessary, silent mutations were introduced to reduce the Cas9 cut of the donor / edited locus, and to avoid homology between the sequence from the insert and the Cas9 cut with the genomic sequence. Guide RNAs were selected to cut as close as possible to the site of insertion and to be predicted as efficient sgRNA (5). PCR mix was: 0.8µl of plasmid template at 50-100ng/µl (standard plasmid miniprep), 2µl of each PCR primers at 100µM, 200µl of 2X Phusion High-Fidelity PCR Master Mix with HF Buffer (NEB, #M0531L), 195.2µl of H2O, and split in 8*50µl in PCR tubes. PCR condition were 98°C / 30s at 98°C, 30s at annealing temperature, 45s at 72°C, for 30 cycles / 10 minutes at 72°C / Hold at 10°C. Annealing temperature was 61.5°C for most PCR repair donors, but sometimes a gradient from 60 °C to 72 °C was performed to obtain single bands on agarose gel. PCR amplicons were pooled and purified together using MinElute PCR Purification Kit (Qiagen, #28004) and eluted in 10µl of H2O. Concentration was measured using a NanoDrop.

Complementary information can be found in Dryad Digital Repository (https://doi.org/10.5061/dryad.63xsj3v5s).

CRISPR/Cas9 KI mix and injection

Dejellied oocytes were fertilized and transferred to a small plastic petri dish, and zygotes were injected with needles containing the CRISPR/Cas9 KI mix (6). Injections were performed until zygotes started dividing. CRISPR/Cas9 KI mix was made fresh by mixing the reagents in the following order at Room Temperature (RT): 1.325µl spyCas9 protein (10-12µg/µl; in 20mM HEPES pH7.5, 500mM KCl, 20% glycerol), 0.83µl KCl 1M, 0.34µl HEPES 0.5M pH7.5, 2.1µl sgRNA (100µM, in 5mM Tris pH7.5), H2O (DNAse/RNAse free, not DEPC treated, Invitrogen, #10977-035) to yield a final volume of 10µl of injection mix, 5pmol of PCR repair donors (in H2O, around 2.5µg for a classic fluorescent protein reporter insertion), 1µl of fluorescent dextran. The injection solution was mixed by pipetting, spun for 5s at 5000rpm in a microcentrifuge, and kept at RT for 5m before loading the injection needles.

spyCas9 protein was prepared as previously described and stored at -80°C (2). sgRNA were chemically synthetized (Sigma, 3nmol, HPLC purified, no modification), reconstituted in 5mM Tris pH7.5 at 100µM, and stored at -80°C. Injection dyes used were dextran Oregon Green (Invitrogen, #D7171, 50µg/µl in H2O), dextran Tetramethylrhodamine (Invitrogen, #D3308, 100µg/µl in H2O), and dextran Texas Red (Invitrogen, #D3328, 100µg/µl in H2O).

After injection, embryos were kept at 17 °C overnight. The day after, they were gently washed twice with 1/3-SW and kept at 17 °C for another day. The following day, embryos were transferred to a new petri dish with 1/3-SW and kept at 27 °C. At day three following injection, injected embryos were selected for positive dextran fluorescence and transferred to a new dish containing 1/3-SW. Positive KI were identified by screening for the corresponding fluorescence in the following days, either live using a Nikon SMZ18 stereomicroscope with appropriate filters; or fixed, mounted, and screened with a confocal microscope. Numbers are (positive F0s / total injected): *lamin* KI (2/23, 5/223, 6/259), *actin* KI (4/23, 4/100, 16/319), *mhc* KI (12/47, 16/95), *cdh1* KI (23/61, 27/131), *sec61b* KI (83/224, 46/149), *rab11a* KI (19/190, 92/426, 28/156). A subset of F0 embryos were examined at high resolution using confocal microscopy and exhibited the expected subcellular localization (4/4 for *lamin* KI, 5/5 for *actin* KI, 8/8 for *mhc* KI, 4/4 for *cdh1* KI, 7/7 for *sec61b* KI, 12/12 for *rab11a*)

For double fluorescent color KI of *mhc*, the injection mix contained each repair donor at half the concentration of what was used for single color KI. Injection dye was 1µl FITC (Invitrogen, #F1300, 0.1mg/ml in Tris 5mM pH7.5) and 1µl dextran A680 (Invitrogen, #D34680, 50ug/µl in H2O). Due to the transient labeling of FITC and the difficulty of identifying

dextran A680 embryos, injected embryos were not sorted. Numbers are (among KI positive F0s): 11/33 mNG+/mScarlet+, 11/33 mNG+/mScarlet-, 11/33 mNG-/mScarlet+ (first experiment) and 5/18 mNG+/mScarlet+, 8/18 mNG+/mScarlet-, 5/18 mNG-/mScarlet+ (second experiment).

KI positive F0s were grown to adulthood and crossed with wild-type animals to determine germline transmission. Numbers are (F0s with germline transmission / positive F0s tested): *actin* KI (2/4), *mhc* KI (1/4), *cdh1* KI (4/4), *sec61b* KI (4/5), *rab11a* KI (2/6). No F0 had germline transmission for *lamin* KI (n>6).

For *mhc*, *cdh1*, *sec61b* and *rab11a* F0s with germline transmission, the % of positive F1s in each brood was determined and was in average 48% (+/- 12) across 19 F0s. Numbers are (positive F1s / total F1s, per positive F0s raised to adulthood): *mhc* KI (35/86 for F0 #1, 0/36 for F0 #2, 0/19 for F0 #3, 0/10 for F0 #4), *cdh1* KI (23/40 for F0 #1, 13/33 for F0 #2, 24/41 for F0 #3, 22/40 for F0 #11), *sec61b* KI (7/14 for F0 #1, 38/59 for F0 #2, 16/91 for F0 #3, 0/20 for F0 #4, 23/50 for F0 #6), *rab11a* KI (0/80 for F0 #1, 21/40 for F0 #2, 0/80 for F0 #3, 0/80 for F0 #4, 32/67 for F0 #5, 0/80 for F0 #6).

Note that in-cross of *cdh1* KI F1s (from F0 #1) gives 72.2% of fluorescent F2s (138/191 F2s), corresponding to what is expected with Mendelian allele transmission from heterozygous animals. Out-cross of *rab11a* KI F2s gives for heterozygous animals approximately half of positive F3s (40/74 and 48/86 for two male F2s) and 100% of positive F3s for homozygous (40/40 and 40/40 for two male F2s)

Complementary information can be found in Dryad Digital Repository (https://doi.org/10.5061/dryad.63xsj3v5s).

Imaging

Embryos were collected and transferred to a well of a 96 well plate containing 1/3-SW. When necessary, primary polyps were anesthetized for 30m-1h by gently adding MgCl2 solution to the 1/3-SW (from a stock solution at 7% w/v of MgCl2 in 1/3-SW, 0.35% final). Next, 1/3-SW (with or without MgCl2) was replaced by a solution of 3 parts of 1X PBS and 1 part of PFA 16% (Pierce, #28908). Fixation was performed for 1-2h at RT with occasional mixing by pipetting. Next, fixed embryos were washed once with 1X PBS, followed by a wash with 1X PBS containing 0.25% Triton X-100, and finally 3 times with 1X PBS. Fixed embryos were transferred to a microscope slide coated with Poly-L-lysine solution (Sigma-Aldrich, #P8920) inside a square of double side tape. Excess 1X PBS was removed, and a drop of VectaShield Antifade Mounting Medium with DAPI (Biozol, #VEC-H-1200-10) was added. Next, a glass coverslip was added, and the slide was sealed with CoverGrip (Biozol, #BOT-23005).

Imaging was performed with a Leica SP8 confocal or a Zeiss LSM880 with appropriate settings for eGFP/mNG and mScarlet imaging. For whole embryos imaging at low resolution, a Biorad Zoe fluorescent microscope was also used. For live imaging of the *mhc::mNG* line, a mesoscopic oblique plane microscope was used to generate maximum intensity projection (600 z slices, 1.3µm apart with 1ms exposure time and 2mW excitation power) (7). For live imaging of *mNG::sec61b* and *eGFP::rab11a* lines, a Zeiss LSM980 Airy Fast microscope was used. Images were processed using ImageJ Fiji and eGFP::Rab11a particles were tracked using Manual Tracking plug-in.

To compare expression of *mhc* between endogenous protein tagging and transgenic expression reporter, *mhc::mNG* line was crossed with a *Prom-mhc::mCherry::sv40-3'UTR* transgenic line (8).

To compare localization of Cdh1::eGFP (Ct eGFP, intracellular) with non-tagged Cdh1, *cdh1::eGFP* line was crossed with wildtype animals, and both eGFP (Torrey Pines Biolabs, #TP401. 1/500 dilution) and Cdh1 proteins (against extracellular sequence, 1/500 dilution) were immuno-stained following conditions previously described (9). Secondary antibodies coupled with A488 and A647 were used (Invitrogen, A-11008 and A-11008) at a dilution of 1/500.

Genotyping and Sequencing

Individual F1s primary polyps were transferred first in a dish containing H2O, and next in PCR tubes. Next, they were lysed in 30µl of QuickExtract DNA Extraction Solution (VWR, #BZYM101098) for 3h at 65 °C (with time-to-time vortexing and spinning), followed by 30min at 100 °C. Finally, 30µl of H2O was added to the lysis, and samples were vortex and stored at -20 °C. Alternatively, adult animals were anesthetized with 0.35% MgCl2, the tentacles were cut and transferred in a dish containing H2O, and next in Eppendorf tubes, spun for 2m at 13000rpm in a microcentrifuge and processed as for individual primary polyps.

For genotyping PCR (with primers to amplify the targeted locus, the 5'/3' insertion junctions, or the fluorescent reporter sequence), the reaction was set up as it was for PCR repair donor, but with 4µl of lysis (2µl for tentacle samples) and a final volume of 20µl. Detection of possible imprecise insertion at the targeted locus, or random insertion of the fluorescent reporter, or formation of long lasting episome, was performed by PCR using fluorescent reporter specific primers on fluorescent-negative F1s (from broods containing also fluorescent-positive F1s). Fluorescent-negative F1s were also sequenced at the targeted locus and NHEJ alleles were identified by the presence of a mix of two sequences in Sanger sequencing (wildtype + NHEJ).

For each primer pair, the best annealing temperature was determined using a temperature gradient from 60 °C to 72 °C, using similar PCR conditions as for synthesis of PCR donors. PCR amplicons were analyzed on agarose gels and imaged using standard UV transilluminator or Typhoon imager (GE Healthcare). PCR amplicons were column purified using QIAquick PCR Purification Kit (Qiagen, #28104), eluted in 30µl H2O, and sent out for Sanger sequencing (Eurofins Genomics). All sequenced insertions were scarless, but eight positive F1s from two *cdh1::eGFP* F0s had a single nucleotide change in the middle of the eGFP sequence (missense mutation without loss of fluorescence), maybe due to error during PCR donor synthesis or during HDR.

Numbers are (scarless insertion in HDR KI allele: 41/41): 1/1 positive F2 pools for *actin* KI; 4/4 positive F1s for *mhc* KI F0#1; 4/4 positive F1s for *cdh1* KI F0#1, 4/4 positive F1s for *cdh1* KI F0#2, 4/4 positive F1s for *cdh1* KI F0#3, 4/4 positive F1s for *cdh1* KI F0#11; 4/4 positive F1s for *sec61b* KI F0#1, 4/4 positive F1s for *sec61b* KI F0#2, 3/3 positive F1s for *sec61b* KI F0#3, 4/4 positiveF1s for *sec61b* KI F0#6; 3/3 positive F1s for *rab11a* KI F0#2; 2/2 positive F1s for *col4* KI F0#1.

Numbers are (NHEJ in non-KI allele: 14/35. From fluorescent negative F1s from F0s with germline transmission of the HDR KI allele): 0/4 negative F1s for *mhc* KI F0#1; 4/4 negative F1s for *cdh1* KI F0#1, 4/4 negative F1s for *cdh1* KI F0#2, 0/4 negative F1s for *cdh1* KI F0#3, 0/4 negative F1s for *cdh1* KI F0#11; 2/4 negative F1s for *sec61b* KI F0#1, 4/4 negative F1s for *sec61b* KI F0#2, 0/3 negative F1s for *sec61b* KI F0#3, 0/4 negative F1s for *sec61b* KI F0#6.

The following PCR primers were used (5' to 3'): GATTCCTATTTGTTTATACTCG, CAATCAAATGCCGGTAAGTTAC, TTCGTCTAGTTTTCTCTAGC, GAGTCTTTCTGACCCATACC for *actin*; TCACCGCTCGGAGATTGTGTCG, AACTAACTGTGCACTTCTCCTG, TAACGTTACCAACTAGGTGG, GACAGATATATTTATGTGGG for *mhc*; AACCAACTAGCCTGAACAAGGC, TACTCTTTAGGATGGCCTTCTG, CAGACGTCGATGATCTGAGC, CTACGACACCTTCCATGTCG for *cdh1*; CGAAAAGTGAAGGCTGCCTACC, GAACACGTCAGTGAAAATGG, AACTATATCTAACTTTATCATG, TTGTGAGTGTAACTTTCTTTGG, CTTTGTCTGAGCTGGCCACCAG for *sec61b*; TTCCGCCAAGTTCCCTGTTG, TGTTCCCTCCCACAACGACG, TCCGATAGGATTGCTGGTTG, CCCGAATCTCCAATAAGTAC for *rab11a*; ACCCTAGAGGCTATCACAATTC, ACCCTGTTAATCCAGGAAAACC, AATTGCCAGGAGATATATTG, AAGAAGTACTTAAATGAGAC for *col4*; GACGTACCCAAATGACAAGACC, GTCTCCAAAGGGGAGGAAG, CTTGTAGAGTTCGTCCATTC, CCATTTCAAGCGGCCATGGTAG, GGTCTTGTCATTTGGGTACGTC, GTGGATACCAAGTACATCGTAC for *mNG*; CGACCACATGAAGCAGCATGAC, GCCATGCCCGAAGGTTATGTAC, TGTATTCCAATTTGTGTCCAAG for *eGFP* (from pAP625 / pAP683); CTTGTACAGCTCGTCCATG, GTGAGCAAGGGCGAGGAGC, CGCCGAGGTGAAGTTCGAGG, GATGCCGTTCTTCTGCTTGTCG for *eGFP* (from pAP1698).

Complementary information can be found in Dryad Digital Repository (https://doi.org/10.5061/dryad.63xsj3v5s).

SUPPORTING MOVIE LEGENDS

Movie S1: Live imaging of a *mhc::mNG* contracting polyp.

Non-anesthetized primary polyp. Data shown in Figure 2B. Scale bar= 100µm.

Movie S2: Live imaging of a *mNG::sec61b* embryos.

Z-stack of a 32-cell stage embryo. Scale bar= 100µm.

Movie S3: Live imaging of a *mNG::sec61b* embryo.

32-cell stage embryo (same as Movie S2). The focus is at the level of the cell cortex and shows ER tubules and patches. Note that some bleaching occurs. Scale bar= 10µm.

Movie S4: Live imaging of a *mNG::sec61b* embryo.

Gastrula stage, with gastrulation hole at the center. Scale bar= 20µm.

Movie S5: Live imaging of a *mNG::sec61b* embryo.

Low resolution of dividing blastomeres (128-cell stage embryo). Scale bar= 20µm.

Movie S6: Live imaging of a *eGFP::rab11a* embryo.

8-cell stage embryo. eGFP::Rab11a forms puncta, possibly corresponding to trafficking vesicles, and some of them were manually tracked (lines). Scale bar= 5µm.

SUPPORTING REFERENCES

1. D. J. Stefanik, L. E. Friedman, J. R. Finnerty, Collecting, rearing, spawning and inducing regeneration of the starlet sea anemone, Nematostella vectensis. *Nat Protoc* **8**, 916–923 (2013).
2. A. Paix, *et al.*, Precision genome editing using synthesis-dependent repair of Cas9-induced DNA breaks. *Proc Natl Acad Sci U S A* **114**, E10745–E10754 (2017).
3. J. A. Gutierrez-Triana, *et al.*, Efficient single-copy HDR by 5' modified long dsDNA donors. *Elife* **7** (2018).
4. B. Zimmermann, *et al.*, Sea anemone genomes reveal ancestral metazoan chromosomal macrosynteny. *bioRxiv* (2020).
5. J. G. Doench, *et al.*, Optimized sgRNA design to maximize activity and minimize off-target effects of CRISPR-Cas9. *Nat Biotechnol* **34**, 184–191 (2016).
6. M. J. Layden, E. Röttinger, F. S. Wolenski, T. D. Gilmore, M. Q. Martindale, Microinjection of mRNA or morpholinos for reverse genetic analysis in the starlet sea anemone, Nematostella vectensis. *Nat Protoc* **8**, 924–934 (2013).
7. R. Singh, *et al.*, An oblique plane microscope for mesoscopic imaging of freely moving organisms with cellular resolution. *bioRxiv* (2022) https:/doi.org/10.1101/2022.07.15.500249.
8. E. Renfer, A. Amon-Hassenzahl, P. R. H. Steinmetz, U. Technau, A muscle-specific transgenic reporter line of the sea anemone, Nematostella vectensis. *Proc Natl Acad Sci U S A* **107** (2010).
9. E. A. Pukhlyakova, A. O. Kirillova, Y. A. Kraus, B. Zimmermann, U. Technau, A cadherin switch marks germ layer formation in the diploblastic sea anemone Nematostella vectensis. *Development (Cambridge)* **146** (2019).
